# A Toolbox of Genetically Encoded FRET-Based Biosensors for Rapid l-Lysine Analysis

**DOI:** 10.3390/s16101604

**Published:** 2016-09-28

**Authors:** Victoria Steffen, Julia Otten, Susann Engelmann, Andreas Radek, Michael Limberg, Bernd W. Koenig, Stephan Noack, Wolfgang Wiechert, Martina Pohl

**Affiliations:** 1 IBG-1, Forschungszentrum Jülich GmbH, 52425 Jülich, Germany; vi.steffen@fz-juelich.de (V.S.); j.otten@fz-juelich.de (J.O.); susann.engelmann@rwth-aachen.de (S.E.); a.radek@fz-juelich.de (A.R.); m.limberg@fz-juelich.de (M.L.); s.noack@fz-juelich.de (S.N.); w.wiechert@fz-juelich.de (W.W.); 2 ICS-6, Forschungszentrum Jülich GmbH, 52425 Jülich, Germany; b.koenig@fz-juelich.de

**Keywords:** FRET-based biosensors, l-lysine detection, metabolite analysis, online monitoring, microbial strain development

## Abstract

**Background:** The fast development of microbial production strains for basic and fine chemicals is increasingly carried out in small scale cultivation systems to allow for higher throughput. Such parallelized systems create a need for new rapid online detection systems to quantify the respective target compound. In this regard, biosensors, especially genetically encoded Förster resonance energy transfer (FRET)-based biosensors, offer tremendous opportunities. As a proof-of-concept, we have created a toolbox of FRET-based biosensors for the ratiometric determination of l-lysine in fermentation broth. **Methods**: The sensor toolbox was constructed based on a sensor that consists of an optimized central lysine-/arginine-/ornithine-binding protein (LAO-BP) flanked by two fluorescent proteins (enhanced cyan fluorescent protein (ECFP), Citrine). Further sensor variants with altered affinity and sensitivity were obtained by circular permutation of the binding protein as well as the introduction of flexible and rigid linkers between the fluorescent proteins and the LAO-BP, respectively. **Results:** The sensor prototype was applied to monitor the extracellular l-lysine concentration of the l-lysine producing *Corynebacterium glutamicum* (*C. glutamicum*) strain DM1933 in a BioLector^®^ microscale cultivation device. The results matched well with data obtained by HPLC analysis and the Ninhydrin assay, demonstrating the high potential of FRET-based biosensors for high-throughput microbial bioprocess optimization.

## 1. Introduction

Nowadays, microbial production of a broad range of commercially interesting compounds, such as vitamins, amino acids, and organic acids is possible [1,2,3,4,5]. To speed up the development of production strains, suitable screening methods are of prime importance. Therefore, specific sensors with an optical readout are of interest to enable the online analysis of target metabolites in a parallelized manner. One option is the use of reporter-based biosensors that translate the presence of a certain metabolite inside the microbial production cell into a fluorescent signal [6]. FRET-based genetically encoded biosensors are also used to sense intracellular metabolite levels in a ratiometric manner [7]. Such sensors were first described in 1997 [8,9] and are now available for a broad range of ligands such as sugars, peptides, and metal ions. For further details the reader is referred to the respective reviews [10,11,12,13]. In FRET-based biosensors of the Venus-flytrap type, the conformational change of the (commonly periplasmic) central binding protein, which occurs upon ligand binding, is translated into an altered FRET-signal of the flanking fluorescent proteins. As FRET-based sensors measure the change of the FRET-ratio between two FRET-partners in response to metabolite binding to a central metabolite binding protein, the signal is independent of the absolute sensor concentration in the cell. Although such sensors are widely used for metabolite analyses in different cells [14], we have recently demonstrated that the FRET-ratio change is influenced by a broad range of parameters that can hardly be controlled inside cells [15]. Thus, in this study we evaluate such sensors for extracellular metabolite analysis specifically for the analysis of l-lysine in culture supernatants of a *C. glutamicum* l-lysine producer.

*C. glutamicum* is a well-known model organism in industrial biotechnology [16] and is used for the industrial large-scale production of amino acids like l-glutamate (2.2 million tons in 2009) or l-lysine (1.5 million tons in 2011) [17]. Modern bioprocess development concepts are followed in Mini Pilot Plants [18], where the amino acid concentrations in the culture supernatants are currently analysed by using either HPLC or the colorimetric Ninhydrin assay [18,19]. However, both methods do not allow for the online detection of l-lysine during cultivation. Another drawback is that the HPLC analysis is time-consuming, making it less suitable for higher throughput screening approaches. By contrast, the Ninhydrin assay is applicable in higher throughput but is not specific for l-lysine. Consequently, there is a demand for a fast and specific l-lysine detection method which can be performed directly in culture supernatants and is also applicable during microtiter plate cultivations, e.g., when using the BioLector^®^ system [20]. To meet all these demands, FRET-based biosensors for l-lysine were developed and validated for optical detection in culture supernatants.

**Figure 1:**
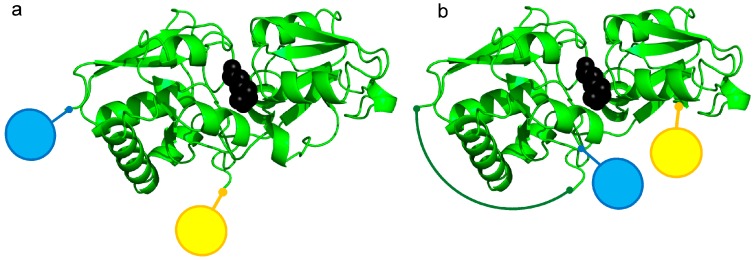
Schematic comparison between the sensor with the native (**a**) and the circular permutated (**b**) l-lysine-l-arginine-l-ornithine binding protein (LAO-BP) with ECFP (blue), Citrine (yellow), bound l-lysine (black) and the N- and C-terminus connecting linker (dark green). The blue and yellow spheres at the N- and C-terminus, respectively, indicate the attached fluorescent proteins.

The sensor was constructed based on the similar work of Okada et al. [21], using the lysine-/arginine-/ornithine-(LAO) binding protein from *Escherichia coli* (*E. coli*) [22], flanked by two fluorescent proteins: the enhanced cyan fluorescent protein (ECFP [23,24]) and a variant of the enhanced yellow fluorescent protein (Citrine [25]). As the protein sequence of the LAO-binding protein from *E. coli* is highly similar to the respective binding protein from *Salmonella typhimurium* (*S. typhimurium*) [26], a similar three-dimensional structure can be assumed. The homology model presented in Figure 1 was obtained based on the crystal structure of the *S. typhimurium* binding protein [27]. Here we describe the construction of a toolbox of highly potent FRET-based l-lysine biosensors based on a circular permutated LAO-binding protein and different linker combinations between the central LAO-binding protein and the flanking fluorescent proteins. One of these sensors was finally successfully validated as an online measurement tool supporting small-scale bioprocess optimization.

## 2. Materials and Methods

### 2.1. Construction of l-Lysine Sensor Plasmids

The first l-lysine sensors were built up from a native (nLAO-BP) [28] or circular permutated lysine-/arginine-/ornithine-binding protein (cpLAO-BP) [21], which were flanked by an enhanced cyan fluorescent protein (ECFP) [23,24] and a variant of an enhanced yellow fluorescent protein (Citrine) [25] in a pRSET vector construct. For construction of the sensor plasmids, the glucose sensor vector pRSET FLII^12^Pglu600µ [29] was used as a template for the pRSET vector construct and the Citrine gene. The genes encoding the ECFP and the binding protein were ordered at GeneArt (Thermo Fisher Scientific, Darmstadt, Germany) (all DNA and protein sequences are shown in the Supplementary Material). The DNA sequence of the permutated binding protein was obtained from the LAO-BP from *E. coli* (*argT*, GenBank No: CP001665.1, 1424860 to 1425642) and was permutated based on the work of Okada et al. [21]. The plasmids of the linker toolbox with protein sequences for a flexible linker: (GGS)_4_ and a rigid linker: KLYPYDVPDYA were created by ligase-free cloning [30,31].

Plasmids were isolated using the QIAprep Spin Miniprep Kit (Qiagen, Hilden, Germany) according to the manufacturer’s recommendation. Amplification of DNA fragments was performed using the DNA polymerase Phusion II and the respective methods ([32], New England Biolabs, Frankfurt am Main, Germany). For plasmid production, *E. coli* DH5α [33] (Thermo Fisher Scientific, Darmstadt, Germany) was used.

### 2.2. Protein Preparation

For production of the l-lysine sensors, *E. coli* BL21 (DE3) [34] cells (Thermo Fisher Scientific, Darmstadt, Germany) were transformed with the respective plasmids and positive clones were selected on agar plates through ampicillin resistance. A pre-culture of 25 mL lysogeny broth medium (LB) [35] containing 100 µg/mL ampicillin was inoculated with an isolated colony of freshly transformed *E. coli* BL21 (DE3) cells and incubated for 16 h at 37 °C. One mL/L of the pre-culture was used to inoculate the main culture consisting of autoinduction medium [36] with 100 µg/mL ampicillin. The culture broth was aliquoted into 500 mL and cultivated in 2 L shaking flasks with two baffles for 2 h (37 °C, 90 rpm) and an additional 48 h at 20 °C and 90 rpm.

As previously described for FRET-based sugar sensors [15], cells were harvested by centrifugation, afterwards disrupted, and the sensor proteins were then purified. Therefore, 20 g of wet cells were resuspended in 80 mL 3-(N-morpholino)propanesulfonic acid (MOPS) buffer (20 mM MOPS, 300 mM NaCl, pH 7.3, with one tablet of cOmplete protease inhibitor (Roche, Basel, Switzerland)) and disrupted by sonication using an UP200s (S3 sonotrode, Hielscher, Teltow, Germany). The sensor proteins carrying an N-terminal hexahistidine tag were purified via immobilized metal chelate affinity chromatography on Ni-NTA agarose (column volume 20 mL, Qiagen, Hilden, Germany, protein detection through multi-wavelength UV-VIS monitor “Monitor UV-900”) by fast protein liquid chromatography (Äkta Purifier, GE Healthcare Life Sciences, Freiburg, Germany). All purification steps were performed at room temperature with a flow rate of 3 mL/min in equilibration buffer (20 mM MOPS buffer, 300 mM NaCl, pH 7.3) and elution buffer with 1 M imidazole. First, washing was performed with equilibration buffer until the baseline was reached again after loading the crude cell extract onto the column. Subsequently, elution of the sensor was performed with a linear imidazole gradient from 0 to 1 M imidazole in 60 column volumes. To desalt the sensor, size exclusion chromatography was performed afterwards. The sensor containing solution was loaded onto a Sephadex G-25 medium (column volume 1 L, GE Healthcare Life Sciences, Freiburg, Germany) and eluted with 20 mM MOPS buffer (pH 7.3). The sensor concentration was adjusted via its absorption maximum at 515 nm to absorption 0.2 in quartz cuvettes, with a 1 cm light path. This absorption equals a protein concentration of 0.18 mg/mL. The purified sensor with adjusted protein concentration was stored in buffer in 1 and 10 mL aliquots in Eppendorf (volume 1.5 mL) and Falcon tubes (volume 15 mL) at 20 °C.

### 2.3. Determination of Binding Isotherms and Dissociation Constants of FRET-Based l-Lysine Sensors in Vitro

The in vitro measurement of binding isotherms was performed in a microtiter plate spectrofluorimeter with a monochromator unit (M-200, Tecan, Männedorf, Switzerland), using 96-well plates with a transparent flat bottom (Nunc F, Thermo Fisher Scientific, Darmstadt, Germany). Per well, 90 µL of sensor solution was mixed with 10 µL of 24 different l-lysine concentrations (final concentrations 0–100 mM) in 20 mM MOPS buffer, pH 7.3. To ensure constant l-lysine concentrations, respective stock solutions containing 0–1 M l-lysine were prepared in 20 mM MOPS buffer (pH 7.3) with adjusted pH and were stored at −20 °C and diluted 1:10. As in previous measurements [15], all data points represent the arithmetic average of 10 measurement cycles of the microtiter plate reader. The standard deviation given for each data point denotes the difference between three independent replicates. ECFP [23,24] as the FRET donor was excited at 428 ± 10 nm, while both fluorescent signals of the FRET partners were recorded at 485 ± 20 nm for ECFP and 515 ± 20 nm for Citrine [25]. The FRET-ratio R is calculated as the quotient of the fluorescence intensities of the acceptor (here: Citrine) and the donor (here: ECFP).

(1) $$R=\frac{I\left(\mathrm{acceptor}\right)}{I\left(\mathrm{donor}\right)}$$

The following characteristic parameters were deduced from binding isotherms: i. the minimal FRET-ratio R_0_; ii. the FRET-ratio at ligand saturation, R_sat_; iii. the sensitivity which represents the difference between the minimal and maximal FRET-ratio ΔR = R_sat_ − R_0_, and the dissociation constant K_d_. K_d_ is the ligand concentration at the inflection point of the S-shaped binding isotherm, referring to the half-maximal FRET-shift. The parameters were estimated using the following equation [15,37]:

(2) $$R=\frac{\Delta R\cdot \left[S\right]}{K_{d}\;+\;\left[S\right]}+R_{0}.$$

The percentage of ΔR from R_0_ was determined as $\Delta R/R_{0}*100\%.$

Fluorescence-independent affinity characterization was performed using isothermal titration calorimetry. The protein solutions in 20 mM MOPS buffer at pH 7.3 were concentrated to values just below their solubility limit resulting in 60 µM for the sensor prototype and 22 µM for the cpLAO-BP, respectively. The measurements were performed in a MicroCal^TM^ iTC_200_ system (GE Healthcare Life Sciences, Freiburg, Germany) at 25 °C with 200 µL of protein solution in the calorimeter cell and 40 µL of l-lysine solution (in 20 mM MOPS buffer, pH 7.3) in the injection syringe. While stirring the cell content at 500 rpm, aliquots of 1 µL or 2 µL were added to the protein solution with 2 s and 4 s per injection, respectively, and a 240 s delay between injections. Due to the different affinities, titrations of cpLAO-BP were conducted with 0.2 mM, 0.15 mM, and 0.1 mM l-lysine solution and titrations of the sensor prototype (00) were performed with 10 mM, 5 mM, and 1 mM l-lysine. As controls, titrations of the respective protein solutions with MOPS buffer without l-lysine and titrations of MOPS buffer with l-lysine were recorded and subtracted from the sensor titration curves in the presence of lysine where appropriate.

### 2.4. Production and Determination of l-Lysine in C. Glutamicum Culture Supernatants

If not stated otherwise, the production of l-lysine was performed similar to that previously described by Unthan et al. [18]. In brief, *C. glutamicum* cultures (wild type [38] or l-lysine producing DM1933 [39]) were inoculated in 1.1-times concentrated CGXII medium [18] with 10 g/L glucose to an OD_600 nm_ = 1 and incubated in 1300 µL aliquots at 30 °C and 1000 rpm in Flowerplates^®^ in a Biolector^®^ cultivation device (m2p-labs GmbH, Baesweiler, Germany). Each row of the 48-well plate contained four wells with culture broth and four wells with in-plate calibration standards, as shown in Section 3.3. The calibration standards were prepared before the cultivation was started using mixtures of the fresh CGXII medium and the culture supernatant of *C. glutamicum strain* DM1933 in various proportions and contained 0, 1, 10, and 100 mM l-lysine (for detailed information see the supporting information, chapter 9). Every 4 h, one row of the Flowerplate^®^ was sampled for later offline analysis using Ninhydrin and HPLC. Therefore 400 µL of culture supernatant was removed, centrifuged, and the supernatant was stored at 4 °C. Subsequently, 100 µL biosensor (prototype) in 20 mM MOPS buffer (pH 7.3) was added to the residual cultivation broth of this particular Flowerplate^®^ row. Due to the six rows of a Flowerplate^®^, six sampling times (0, 4, 8, 12, 16, and 20 h) could be monitored in one cultivation cycle. The following online parameters were recorded during the cultivation: pH, pO_2_, biomass formation (backscatter), and two fluorescence signals (λ_Ex_ = 430 ± 5 nm, λ_Em_ = 468 ± 5 nm, λ_Ex_ = 430 ± 5 nm and λ_Em_ = 532 ± 5 nm).

The offline determination of l-lysine was performed by HPLC as described by Limberg et al. [40] and using the Ninhydrin assay as described by Unthan et al. [18].

## 3. Results and Discussion

### 3.1. Construction of FRET-Based Biosensors without and with Circular Permutation of the LAO-Binding Protein

The first sensors were constructed with the native LAO-BP, which belongs to the type II [41] and cluster F [42] periplasmic binding proteins, where both termini are localized on the N-terminal domain of the two globular domains. Consequently, fusion of the native lysine binding protein (nLAO-BP) with the two fluorescent proteins ECFP and Citrine also resulted in the localisation of both fluorescent proteins on the same globular domain of the binding protein (Figure 1a). Therefore, only a small change in orientation and distance of the FRET donor and acceptor was anticipated, which would result in a rather weak FRET-ratio shift upon binding of l-lysine as was also earlier demonstrated by Okada et al. [21]. Using a permutated LAO-binding protein (cpLAO-BP), the new termini were located on each of the two globular domains, thus enabling a better translation of the conformational change of the binding protein upon metabolite binding to the relative orientation of the fluorescent proteins (Figure 1b). The circular permutation was performed through deletion of one of the two polypeptide linkers between the two domains and connection of the former N- and C-termini via a flexible linker sequence (GGS)_4_ (as indicated in dark green in Figure 1b).

**Figure 2:**
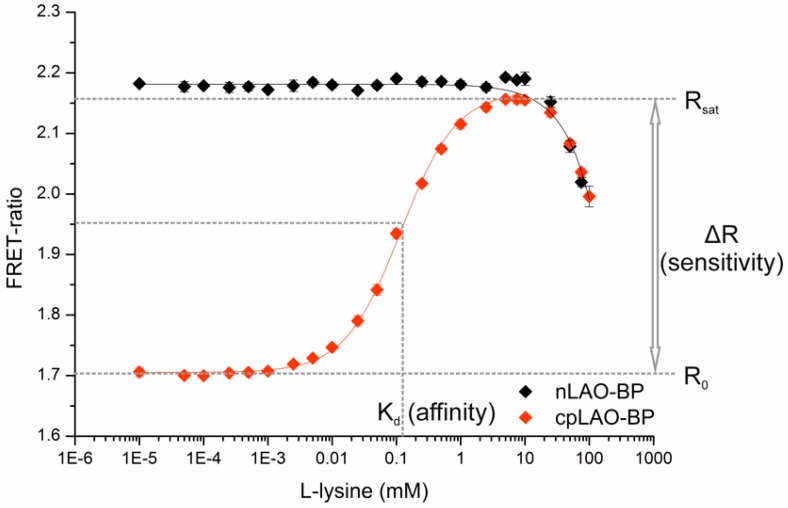
Binding isotherms of the two sensors constructed with the native (nLAO-BP, black) and the circular permutated (cpLAO-BP, red) LAO binding protein. Also indicated are the readout parameters R_0_, R_sat_, K_d_ (corresponding to the affinity), and ΔR (as a measure for the sensor sensitivity).

After purification, binding isotherms with l-lysine were recorded for both sensor constructs. As expected, the sensor with the nLAO-BP displayed a stable FRET-ratio at 2.18 and no FRET-ratio shift (Figure 2), which was in line with the results from Okada et al. [21]. By contrast, the sensor with the cpLAO-BP showed a typical sigmoidal binding isotherm with a high sensitivity, which referred to a FRET-ratio shift of ΔR = 0.47 (28%, Figure 2). Similar sensors described in the literature displayed a FRET-ratio shift of 57% [21] or of ΔR = 0.28 (56%) [43], respectively.

A dissociation constant of K_d_ = 107 ± 5 µM for l-lysine was estimated for the sensor with the cpLAO-BP (Figure 2), whereas the single lysine binding protein derived from *E. coli* was described with a K_d_ of 3 µM, measured by equilibrium dialysis [22]. Thus, the affinity of the sensor construct with the cpLAO-BP was reduced by a factor of 36, which was most likely caused by the circular permutation of the binding protein and further by the fused fluorescent proteins. Additionally, the affinity for l-arginine (K_d_ ca. 25 µM), as well as l-histidine (K_d_ > 100 mM) and l-glutamine (as a weakly binding and non-binding control) were tested (SI, Figure S1), demonstrating that the dissociation constants for lysine and arginine are still on the same order of magnitude. In the literature, respective affinities were determined by equilibrium dialysis for the LAO-binding protein of *E. coli* without permutation and without flanking fluorescent proteins in a different buffer (10 mM KPi buffer, pH 7.0, with 50 mM NaCl), resulting in highly similar dissociation constants for arginine (K_d(Arg)_ = 1.5 µM), lysine (K_d(Lys)_ = 3.0 µM), and ornithine (K_d(Orn)_ = 5.0 µM) [22]. The same is true for the LAO-binding protein from *S. typhimurium*, where dissociation constants for arginine (K_d(Arg)_ = 14 nM) and lysine (K_d(Lys)_ = 15 nM) were available from equilibrium dialysis in 10 mM NaPi buffer, pH 7.0. In this case, the K_d_ for histidine was also measured (500 nM), exceeding those for arginine and lysine by a factor of 30 [26].

The decrease of the FRET-ratio with the four highest tested l-lysine concentrations (Figure 2) could be explained by a pH-shift as a result of the 1:10 dilution of the (pH-adjusted) highly concentrated l-lysine stock solution in buffer. pH-effects on the fluorescent proteins and similar FRET-based biosensors have also been described previously [15]. As demonstrated in Figure S2, all relevant parameters of the binding isotherm (R_0_, R_sat_, K_d_) were influenced by the pH.

### 3.2. Construction of a Sensor Toolbox Using Flexible and Rigid Linkers

After successful construction of a highly sensitive sensor based on the cpLAO-BP (Figure 2), a sensor toolbox was built to further improve the sensitivity (FRET-ratio change) of the sensor prototype. A high sensitivity in buffer is needed to achieve a readable signal in complex matrices such as fermentation broths, where the presence of further metabolites are likely to quench the signal intensity [15].

**Figure 3:**
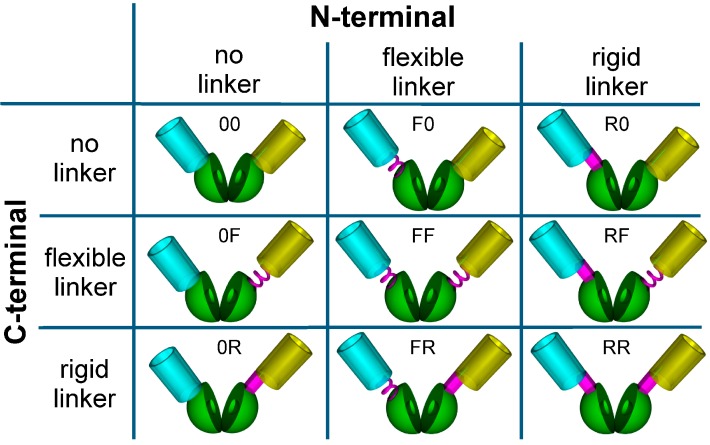
Toolbox of nine different Förster Resonance Energy Transfer (FRET)-based biosensor constructs based on the cpLAO-BP (green) from *E. coli*, using ECFP (blue) as a FRET donor, and Citrine (yellow) as a FRET acceptor. The linker sequences between the fluorescent proteins and the binding protein are shown in magenta. The spring symbolizes a flexible linker (F: (GGS)_4_), the block stands for a rigid linker (R: KLYPYDVPDYA), 0: no linker. The abbreviations for the nine different sensor constructs used in the text are shown next to their respective pictograms.

The toolbox encompassed eight additional biosensors obtained by combinations of a flexible [44,45] ((GGS)_4_) and a rigid [46] (KLYPYDVPDYA) linker, placed between the cpLAO-BP and the fluorescent proteins (Figure 3).

**Figure 4:**
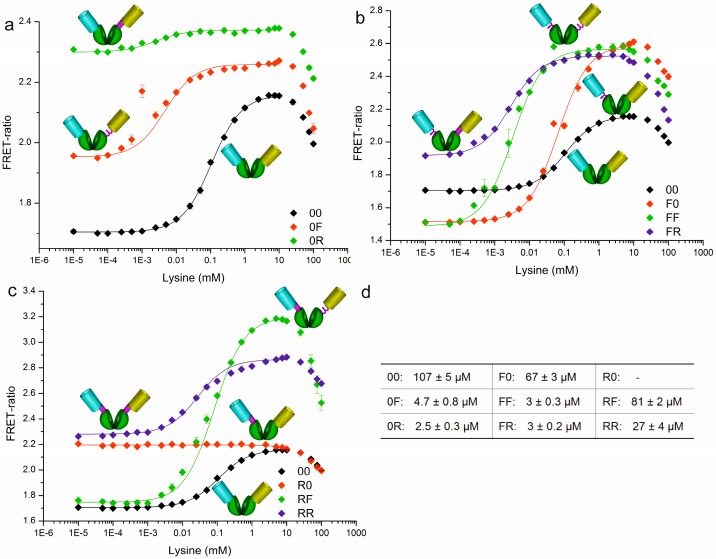
Binding isotherms of the nine different sensor toolbox variants. Next to the curves, the pictograms of the sensor constructs are shown. In all sections, the construct without additional linkers is shown in black. (**a**), the sensor variants without an N-terminal linker construct are presented; (**b**) the sensor variants with an N-terminal flexible linker construct are shown; (**c**) the sensor variants with an N-terminal rigid linker are shown; (**d**) the K_d_-values for l-lysine of all constructs are listed.

After engineering, production, and purification, the nine sensor constructs were first characterized in MOPS buffer. The respective binding isotherms are shown in Figure 4. It is obvious that the linkers do not only influence the sensitivity (FRET-ratio shift) of the sensors but also their affinity (K_d_). An altered sensitivity was expected depending on the flexibility of the linkers as they can decrease or increase the degrees of freedom for the fluorescent proteins. The influence on the affinity, however, was unexpected because the amino acid residues forming the binding pocket of the LAO-BP were not altered by the linkers or by the circular permutation. In order to visualize the effects induced by the linkers, three different clusters of sensors are shown in Figure 4.

The constructs with an additional C-terminal linker relative to the linker-free prototype (black) are shown in Figure 4a. A C-terminal linker increased the affinity while the sensitivity was decreased. This is especially notable for the 0R-sensor with one C-terminal rigid linker (Figure 4a), which shows a K_d_ of 2.5 µM for l-lysine and a decreased sensitivity of 0.07. The 0R-sensor is thus 43 times more affine and 6.6 times less sensitive compared to the prototype.

The constructs with an N-terminal flexible linker compared to the linker-free prototype (black) are shown in Figure 4b. An N-terminal flexible linker increased the sensitivity from 0.46 for the prototype up to 1.1 (2.4 times higher). The biosensors with the highest affinities were obtained with two linkers: a 36-fold increase in affinity was observed (for FF and FR: K_d_ = 3 µM vs. 107 µM for the prototype).

Addition of a rigid N-terminal linker also improved the affinity and varied the sensitivity (Figure 4c). Accordingly, the RF-sensor with a rigid N-terminal linker and a flexible C-terminal linker showed the highest sensitivity of 1.46, which is 3.2-fold higher compared to the prototype. This sensor displayed a K_d_ of 81 µM, which is almost the same range as the sensor prototype.

In summary, the flexible and rigid linkers led to biosensors with varied sensitivity (which was expected), but also altered affinity (which was not expected). It can be assumed that the observed influence on the sensitivity is most likely due to the spacer effect influencing the degrees of freedom for the fluorescent proteins. Thereby, rigid and flexible linkers can cause different effects.

One rigid linker rendered the sensor almost (0R) or completely (R0) dysfunctional. Introduction of only one C-terminal linker decreased the sensitivity, while an N-terminal linker increased the sensitivity. This holds especially true for the flexible linker.

All linkers increased the affinity towards l-lysine. The observed effects can be partly explained by the special conformation of the circular permutated lysine binding protein. Due to the circular permutation, the termini of the LAO-BP most probably adjoin directly to the binding pocket and thus any fusions (linker, fluorescent proteins) might influence the binding affinity directly or indirectly. With all linker combinations, the dynamic range for l-lysine quantification could be improved from 20 µM to 2 mM (only prototype) to a lysine concentration range of 0.3 µM to 2 mM when applying the whole toolbox.

Isothermal titration calorimetry (ITC) was used to determine the influence of the flanking fluorescent proteins on the affinity of the binding protein towards l-lysine. Quantitative interpretation of ITC data in terms of binding model and affinity constants requires data recorded with protein concentration in the cell exceeding the dissociation constant of the interaction by a safe margin. This was especially challenging for the sensor prototype with a solubility limit of 60 µM. All binding isotherms derived from the titration of 22 µM of binding protein (cpLAO-BP) in the cell with three different l-lysine concentrations (0.2 mM, 0.15 mM, and 0.1 mM) could be fitted to the model of a single binding site, i.e., a one-to-one complex of binding protein and l-lysine, characterized by a dissociation constant K_d_ = 1.5 ± 0.3 µM, N = 0.35 ± 0.05 binding sites per protein molecule, and a binding enthalpy ΔH = −55 ± 3 kcal·mol^−1^. An exemplary ITC data trace and the derived binding isotherm of this interaction are depicted in Figure S2 of the Supplementary Materials. The low value of N most likely indicates that about two thirds of the binding proteins are unable to bind l-lysine.

ITC data traces recorded with 60 µM sensor prototype in the measuring cell and three different l-lysine concentrations (10 mM, 5 mM, and 1 mM) in the syringe indicated that the binding of l-lysine to the sensor is at least one order of magnitude weaker than the l-lysine binding to cpLAO-BP. Raw data obtained in one of the titrations of the sensor protein with l-lysine are shown in Figure S3. The observed enthalpy change per mole of injected l-lysine is significantly smaller for the sensor protein in comparison to the binding domain alone. A meaningful quantitative fit of the binding isotherm of such a weak interaction would require ITC traces recorded with at least a ~500 µM protein concentration or more. Such data could not be obtained due to the limited solubility of the sensor protein.

Comparison of the ITC data recorded with cpLAO-BP and the FRET data acquired for nine different sensor variants (see Figure 4d) clearly show a higher affinity of the individual binding domain for l-lysine (K_d_ = 1.5 ± 0.3 µM). Reduction of l-lysine affinity due to the addition of the two fluorescent domains is also reflected by the ITC data on the sensor prototype. Our data indicate that the fluorescent protein domains influence the structure and/or flexibility of the binding protein and/or the access to the ligand binding site. The observation that rigid or flexible linkers between the central cpLAO-BP and the fluorescent proteins invert this effect partially, always yielding in biosensors with higher affinity compared to the prototype, supports this interpretation (Figure 4). Particularly, combinations with flexible linkers generate sensors with dissociation constants up to 2.5 to 5 µM, which is in the same affinity range as the binding protein without fluorescent proteins (K_d_ = 1.5 ± 0.3 µM). In summary, all constructs with linkers showed a higher affinity for l-lysine than the sensor prototype and resulted in a sensor toolbox with K_d_-values ranging from 2.5 to 107 µM.

### 3.3. Development of a Measurement Protocol Applying the FRET-Based Biosensors

**Figure 5:**
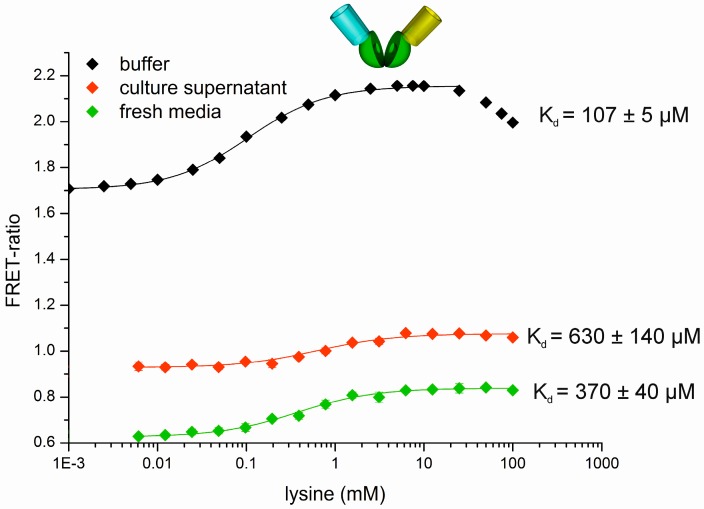
Comparison of binding isotherms of the sensor prototype (00) in 20 mM 3-(N-morpholino)propanesulfonic acid (MOPS) buffer (black), in culture supernatant, and in fresh CGXII-medium, respectively. The curve in 20 mM MOPS buffer, pH 7.3 (black) was recorded in 96-well plates in a microtiter plate spectrofluorimeter (M-200, Tecan, Männedorf, Switzerland). 90 µL sensor solution (in 20 mM MOPS buffer, pH 7.3, sensor concentration OD_515 nm_ = 0.2 equals 0.18 mg/mL) and 10 µL of the respective l-lysine stock solution (10×) were used to achieve the given l-lysine concentration by a 1:10 dilution. The calibration in fresh CGXII-medium with 10 g/L glucose [47] and culture supernatant of a *C. glutamicum* wildtype culture [38] in the stationary phase were recorded in Flowerplates^®^ directly in the BioLector^®^ cultivation device. 900 µL medium (pH 7, green) or culture supernatant (pH 7.5, red) and 100 µL sensor solution (in 20 mM MOPS buffer, pH 7.3, sensor concentration OD_515 nm_ = 0.4 equals 0.36 mg/mL) were used.

Subsequently, the sensor prototype (00, Figure 3) was tested as an online tool for bioprocess development at the microscale. As we have reported earlier for similar FRET-based sensors for glucose and maltose, the FRET-ratio change, which corresponds to the sensitivity of such sensors, is quenched in more complex matrices than buffer and pH changes can influence the affinity [15]. As demonstrated in Figure 5, this also holds true for the l-lysine sensor prototype. It can clearly be seen that the increasing complexity of the solvent from i. buffer over ii. fresh CGXII medium to iii. medium obtained from the stationary phase quenched the signal intensity and shifted the apparent K_d_ for l-lysine to higher concentrations. The latter case is most probably related to pH changes, since the pH changed from 7.0 (fresh CGXII medium) to pH 7.5 in the stationary phase.

**Figure 6:**
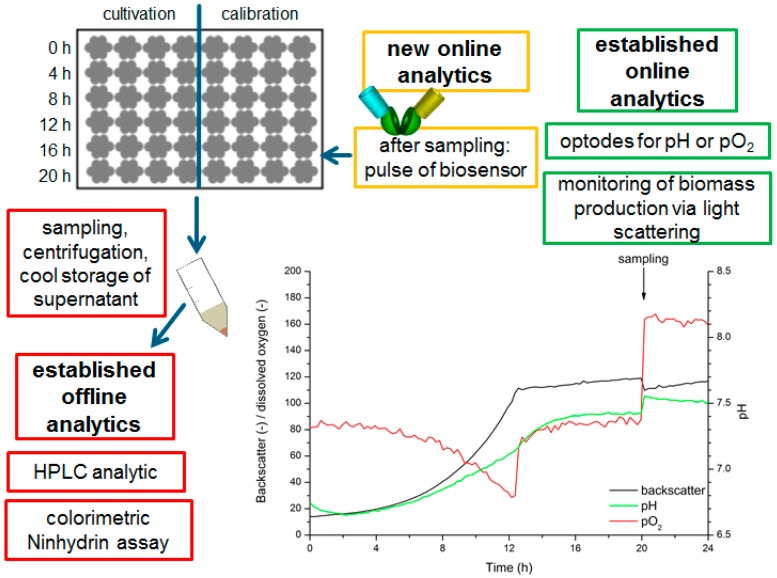
Workflow for cultivation of l-lysine producing *C. glutamicum* with in-plate calibration: Overview of the cultivation and sampling process including the online and offline analytics. The cells were cultivated in one halve of the wells of a Flowerplate^®^ while the other halve was reserved for calibration standards. Online analytics were performed during the entire cultivation time with optodes on the bottom of the well (measurement of pH and pO_2_) and with measurement of scattered light for observation of the biomass formation. The cultivation of l-lysine producing *C. glutamicum* DM1933-cells was performed in 24 identically inoculated wells of a Flowerplate^®^. At each sampling point, one row of the plate was sampled and subsequently the biosensor solution (in 20 mM MOPS buffer, pH 7.3, sensor concentration OD_515 nm_ = 0.4 equals 0.36 mg/mL) was added. In the offline analytics l-lysine concentrations were determined with HPLC and the colorimetric Ninhydrin assay.

To compensate for these effects, a simplified calibration protocol was established directly in the BioLector^®^ Flowerplate^®^, in which the calibration standards for each time point were prepared to enable the estimation of the lysine concentration. The best results were obtained when the measurements were performed in the first 20 min after addition of the sensor to the culture supernatant. Therefore, a pulsing strategy was developed to determine the l-lysine concentrations directly in the culture supernatants of l-lysine producing cultures. The corresponding experimental setup is illustrated in Figure 6. In this setup, one Flowerplate^®^ was divided into two parts: In one part the producing cells are cultivated in several wells, and in the other part the in-plate calibration of the sensor prototype was performed. The cultivation was monitored online with optodes for pH and pO_2_ determination fixed at the bottom of the Flowerplate^®^, while the biomass formation was monitored via scattered light. Every four hours, a sample (400 µL) from each culture well was taken and stored at 4 °C for further offline analytics and 100 µL of sensor solution was added (0.36 mg/mL in MOPS buffer 20 mM, pH 7.3). Per sample time point, one row of the Flowerplate^®^ (four replicates) was sampled (for details see SI chapter data analysis). The fluorescent signals were detected directly in the Biolector^®^ cultivation device and finally compared with the results obtained by the established offline analytical systems (HPLC, Ninhydrin assay) [18].

As a result, the l-lysine concentrations determined via FRET-based measurements were in the same concentration range as those determined by the Ninhydrin assay and HPLC, respectively, (Table 1). As already shown in Figure 5, the FRET-ratios differ due to different medium components at the beginning and at the end of the cultivation. However, due to the in-plate calibration it was possible to directly estimate the l-lysine concentration in the wells of the Flowerplate^®^, which was confirmed by the analytical offline methods. This worked with sufficient reliability until a l-lysine concentration of about 3–10 mM was reached (Table 1).

The upper detection limit in the present system is assumed to be between 10 and 100 mM (cf. Figure 5 and Figure S5). The system was not sensitive enough to detect l-lysine concentrations between 0 and 1 mM lysine. Besides the varying medium composition during the growth of *C. glutamicum*, the presence of the cells during measurements with the sensor may also influence the signal relative to the cell-free calibration (see Figure 5 and Figure S5 (SI)). Additionally to pH-changes, auto-fluorescence of the medium could also disturb the signal. However, in our system auto-fluorescence was ca. 25 times less intense than the fluorescent signal mediated by the sensor (e.g., 0.4 versus 11, see SI Table S4, A01, ECFP signal).

One further explanation for the deviation from the calibration curve (Figure 5) could be that there were other amino acids in the culture medium competing with lysine for the binding site. As was determined earlier by S. Noack and coworkers, the extracellular metabolite concentration in the culture broth of a lysine producing *C. glutamicum* strain (DM1800) contains a broad range of different amino acids, as well as several other metabolites that are secreted in low amounts during cultivation [48]. In particular, besides l-lysine, l-arginine is specifically bound by the cpLAO-BP. However, by taking the different strain background and measured concentration profiles in this study (e.g., l-arginine only accumulates to 35 µM after 50 h of substrate limitation) of the former study [48] into account, we would rather exclude any competing effects of such “by-products” on the biosensor performance under our experimental conditions.

Taken together, the mechanistic understanding of the different, potentially combined, influencing factors on the presented FRET-based biosensors requires much more detailed investigation that is far beyond the scope of this study, but is highly interesting for future works.

Although the estimation of l-lysine concentrations with FRET-based biosensors is not yet as accurate as estimates made with the Ninhydrin assay or the HPLC method, it has several advantages. The main advantage is the possible estimation of l-lysine concentrations directly in the Flowerplate^®^ during the cultivation. Furthermore, the biosensor-based measurement can be performed simultaneously in several wells while HPLC analysis needs at least a 20 min measurement time per sample. The Ninhydrin assay is equally suited for high-throughput applications, but it detects all primary amines and can only be applied as an endpoint determination. Finally, a FRET-based l-lysine biosensor can principally be applied for online measurements in culture supernatants.

## 4. Conclusions

We have successfully demonstrated that FRET-based biosensors are reliable tools for the determination of metabolite concentration directly in culture supernatants. In the present case, a new l-lysine sensor was tested as a proof-of-concept in a Biolector^®^ cultivation device for rapid l-lysine detection. This concept can be expanded in several ways: i. Depending on the concentration threshold to be detected, biosensor variants with different affinities can be applied, e.g., generated through linker combinations as demonstrated in Figure 4. ii. Other metabolites can be sensed using respective binding proteins. There is already a wide variety of binding proteins known [42] and sensors for numerous ligands are already available or can be constructed. iii. Combinations of more than one sensor or more than one sensor concept can be realised using different fluorescent proteins. Two main challenges have to be addressed to further develop the application of FRET-based biosensors for in vitro analysis: future variants must be less pH sensitive and should show an even higher FRET-ratio that provides a better signal-to-noise ratio. pH sensitivity is predominantly caused by the fluorescent proteins [15], thus we are currently evaluating other fluorescent proteins to reduce this problem and to ease the calibration of measurements in complex culture supernatants.

## Figures and Tables

**Table 1:** l-Lysine concentrations in culture supernatants of *C. glutamicum* DM1933. Samples were taken every 4 h and l-lysine concentrations were determined with the biosensor prototype solution, the Ninhydrin assay, and the HPLC method. For further details, please see the legend of Figure 6.

Time	FRET-Ratio	l-Lysine FRET	l-Lysine Ninhydrin	l-Lysine HPLC
0 h	0.69 ± 0.01	0-1 ± 0.12 mM	0 ± 0 mM	0.04 ± 0.01 mM
4 h	0.68 ± 0.01	0-1 ± 0.01 mM	1.6 ± 0.1 mM	0.7 ± 0.01 mM
8 h	0.73 ± 0.01	3 ± 0.01 mM	2.8 ± 0.2 mM	3.0 ± 0.1 mM
12 h	0.78 ± 0.01	15 ± 0.02 mM	8.5 ± 0.3 mM	9.6 ± 0.5 mM
16 h	0.78 ± 0.01	15 ± 0.3 mM	12.2 ± 0.8 mM	13.2 ± 0.9 mM
20 h	0.76 ± 0.01	12 ± 0.04 mM	14.6 ± 2.6 mM	12.3 ± 0.4 mM

## Supplementary Materials

The supplementary materials are available online at http://www.mdpi.com/1424-8220/16/10/1604/s1.

## Abbreviations

cpLAO-BP	circular permutated lysine-/arginine-/ornithine-binding protein
ECFP	enhanced cyan fluorescent protein
HPLC	high-performance liquid chromatography
FRET	Förster resonance energy transfer
ITC	isothermal titration calorimetry
LAO	l-lysine-/l-arginine-/l-ornithine-
nLAO-BP	native l-lysine-/l-arginine-/l-ornithine-binding protein
R_0_	minimal FRET-ratio
R_sat_	saturated FRET-ratio
