# Supplementary material for: A Toolbox of Genetically Encoded FRET-Based Biosensors for Rapid l-Lysine Analysis
Source: Sensors (Basel). 2016 Sep 28;16(10):1604. doi: 10.3390/s16101604 (PMC5087393; doi:10.3390/s16101604)
Supplement: Supplementary file 1 [file sensors-16-01604-s001.pdf]

Supplementary Materials: A Toolbox of Genetically Encoded FRET-Based Biosensors for Rapid L-Lysine Analysis

Victoria Steffen, Julia Otten, Susann Engelmann, Andreas Radek, Michael Limberg, Bernd W. Koenig, Stephan Noack, Wolfgang Wiechert and Martina Pohl

1. Titration Curves of the Sensor Prototype with L-lysine, L-arginine, L-histidine, and L-glutamine

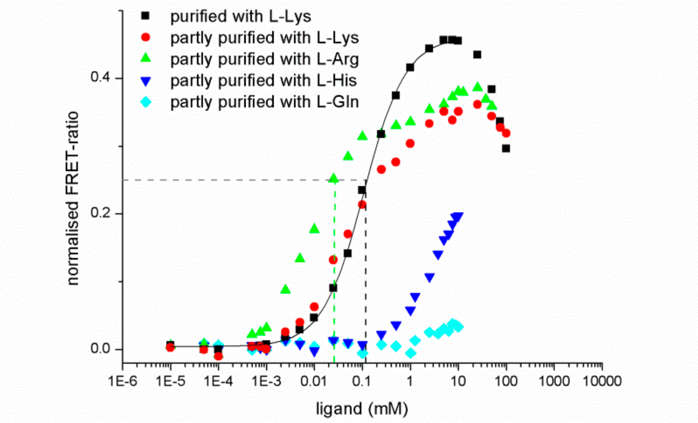

Figure S1. Binding isotherms of the sensor prototype with L-lysine, L-arginine, L-histidine, and L-glutamine. Distortion of the titration curve >0.1 mM L-arginine and L-lysine, respectively, is due to the use of a crude sensor preparation. The results demonstrate that the sensor with the cpLAO-binding protein still has similar affinities for L-lysine (107 μM) and L-arginine (ca. 25 μM), whereas the affinity for L-histidine is much lower (>100 mM).

2. pH-Dependent Binding Isotherms of the Sensor Prototype

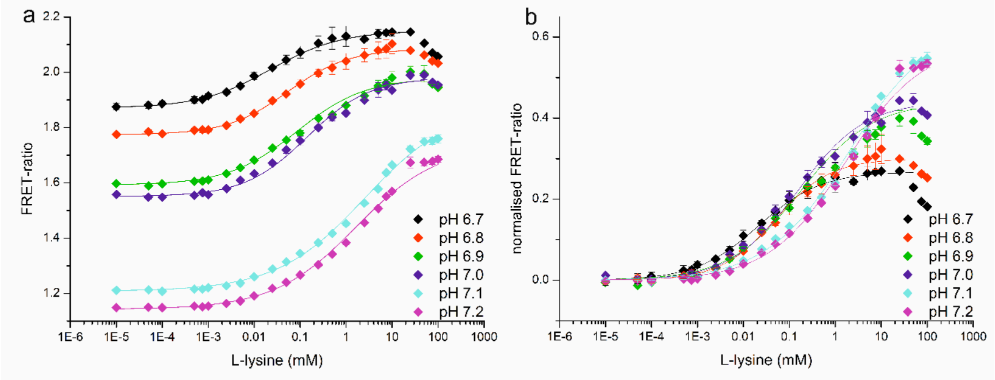

Figure S2. Binding isotherms of the sensor prototype in MOPS buffer with pH-values between pH 6.7 to 7.2. The solution of the sensor prototype was stored in 20 mM MOPS buffer, pH 7.3. This buffer was replaced by 20 mM MOPS buffer with the respective target pH via ultrafiltration. The recorded binding isotherms are shown in (**a**) and for better comparison the normalized binding isotherms are shown in (**b**).

3. Isothermal Calorimetry

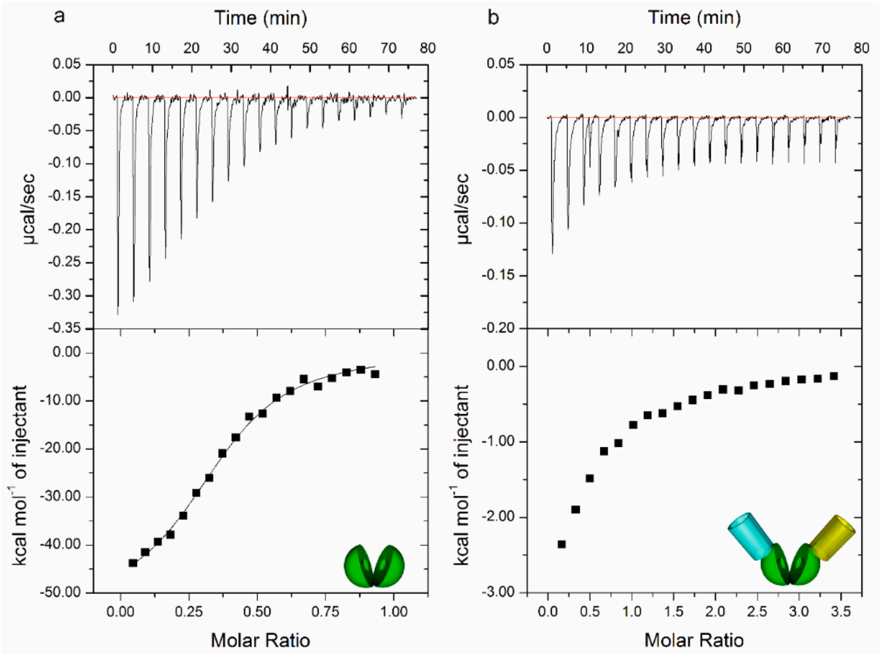

Figure S3. Isothermal titration calorimetry data reflecting L-lysine binding to the binding protein cpLAO-BP (**a**) and the complete sensor prototype containing this binding protein (**b**). The upper two panels show baseline subtracted raw data for titration of 22 µM cpLAO-BP with 100 µM L-lysine (left) and 60 µM sensor prototype with 1 mM L-lysine (right). The lower panels display enthalpy changes per added mole of L-lysine as a function of the total molar ratio of lysine to protein in the calorimeter cell, reflecting binding isotherms. Only the sigmoidal isotherm in the left panel can be reliably fit to a binding model (single binding site, K_d = 1.5 µM, N = 0.36, ΔH = -58 kcal mol^{-1}). Please note the different scaling of the y-axis in (**a**) and (**b**).

4. Protein Sequences from *S. typhimurium* and *E. coli*

S. typhimurium ALPQTVRIGT DTTYAPFSSK DAKGEFI GFD IDLGNEMCKR MQVKCTWVAS DFDALIPSLK
E. coli ALPETVRIGT DTTYAPFSSK DAKGDFVGFD IDLGNEMCKR MQVKCTWVAS DFDALIPSLK

S. typhimurium AKKIDAIISS LSITDKRQQE IAFSDKLYAA DSRLIAAKGS PIQPTLESLK GKHVGVLQGS
E. coli AKKIDAIISS LSITDKRQQE IAFSDKLYAA DSRLIAAKGS PIQPTLDSLK GKHVGVLQGS

S. typhimurium TQEAYANDNW RTKGVDVVAY ANQDLI YSDL TAGRLDAALQ DEVAASEGFL KQPAGKEYAF
E. coli TQEAYANETW RSKGVDVVAY ANQDLVYSDL AAGRLDAALQ DEVAASEGFL KQPAGKDFAF

S. typhimurium AGPSVKDKKY FGDGTGVGLR KDDTELKAAF DKALTELRQD GTYDKMAKKY FDFNVYGD
E. coli AGSSVKDKKY FGDGTGVGLR KDDAELTAAF NKALGELRQD GTYDKMAKKY FDFNVYGD

Figure S4. Comparison of the protein sequences derived from *S. typhimurium* and *E. coli*. The lysine binding proteins show high similarity. Homologous substitutions are marked in green, and non-homologous substitutions are marked in red. The amino acids involved in lysine binding are underlined. The sequence deleted during the circular permutation is marked in yellow.

5. Characteristic Mutations of the Fluorescent Proteins Relative to GFP

ECFP: F64L/S65T/Y66W/N146I/M153T/V163A [1]
Citrine: S65G/V68L/Q69M/S72A/T203Y/H231L [2]

6. DNA and Protein Sequence of the Sensor Construct with cpLAO-BP

The DNA sequence encoding the His-Tag is highlighted in **bold**, the sequence of CFP is marked blue, the recognition site of the restriction enzymes is underlined, the LAO-binding protein sequence is shown in green, and the Citrine sequence is shown in yellow. The same code was used for the protein sequence.

6.1. Nucleotide Sequence:

ATGCGGGGTTCT**CATCATCATCATCATCAT**GGTATGGCTGATACTCGCATTGGTGTAAC
AATCTATAAGTCGGCTGGTATGGTGAGCAAGGGCGAGGAGCTGTTCACCGGGGTGGTGCCC
ATCCTGGTCGAGCTGGACGGCGACGTAAACGGCCACAAGTTCAGCGTGTCCGGCGAGGGC
GAGGGCGATGCCACCTACGGCAAGCTGACCCTGAAGTTCATCTGCACCACCGGCAAGCTG
CCCGTGCCCTGGCCCACCCTCGTGACCACCCTGACCTGGGGCGTGCAGTGCTTCAGCCGCT
ACCCCGACCACATGAAGCAGCACGACTTCTTCAAGTCCGCCATGCCCGAAGGCTACGTCCA
GGAGCGCACCATCTTCTTCAAGGACGACGGCAACTACAAGACCCGCGCCGAGGTGAAGTT
CGAGGGCGACACCCTGGTGAACCGCATCGAGCTGAAGGGCATCGACTTCAAGGAGGACGG
CAACATCCTGGGGCACAAGCTGGAGTACAACTACATCAGCCACAACGTCTATATCACCGCC
GACAAGCAGAAGAACGGCATCAAGGCCAACTTCAAGATCCGCCACAACATCGAGGACGG
CAGCGTGCAGCTCGCCGACCACTACCAGCAGAACACCCCCATCGGCGACGGCCCCGTGCT
GCTGCCCGACAACCACTACCTGAGCACCCAGTCCGCCCTGAGCAAAGACCCCAACGAGAA
GCGCGATCACATGGTCCTGCTGGAGTTCGTGACCGCCGCCGGGATCGGATCCGGCACCGGT
GTAGGGCTACGTAAAGATGATGCTGAACTGACGGCTGCCTTCAATAAGGCGCTTGGCGAGC
TGCGTCAGGACGGCACCTACGACAAGATGGCGAAAAAGTATTTCGACTTTAATGTCTACGG
TGACGGTGGCAGTGGAGGGAGCGGTGGAAGTGGCGGAAGCGCGCTACCGGAGACGGTAC
GTATCGGAACCGATACCACCTACGCACCGTTCTCATCGAAAGATGCTAAAGGTGATTTTGTT
GGCTTTGATATCGATCTCGGTAACGAGATGTGCAAACGGATGCAGGTGAAATGTACCTGGG
TTGCCAGTGACTTTGACGCGCTGATCCCCTCACTGAAAGCGAAAAAAATCGACGCTATTAT
TTCGTCGCTTTCCATTACCGATAAACGTCAGCAGGAGATTGCCTTCTCCGACAAGCTGTACG
CCGCAGATTCTCGTTTGATTGCGGCCAAAGGTTCACCGATTCAGCCAACGCTGGATTCACTG
AAAGGTAAACATGTTGGTGTGCTGCAGGGATCAACCCAGGAAGCTTACGCTAACGAGACC
TGGCGTAGTAAAGGCGTGGATGTGGTGGCCTATGCCAACCAGGATTTGGTCTATTCCGATCT
GGCTGCAGGACGTCTGGATGCTGCGTTACAAGATGAAGTTGCTGCCAGCGAAGGATTCCTC
AAGCAACCTGCTGGTAAAGATTTCGCCTTTGCTGTCGACGAGCTGTTCACCGGGGTGGTGCC
CATCCTGGTCGAGCTGGACGGCGACGTAAACGGCCACAAGTTCAGCGTGTCCGGCGAGGG
CGAGGGCGATGCCACCTACGGCAAGCTGACCCTGAAGTTCATCTGCACCACCGGCAAGCT
GCCCGTGCCCTGGCCCACCCTCGTGACCACCTTCGGCTACGGCCTGATGTGCTTCGCCCGCT
ACCCCGACCACATGAAGCAGCACGACTTCTTCAAGTCCGCCATGCCCGAAGGCTACGTCCA
GGAGCGCACCATCTTCTTCAAGGACGACGGCAACTACAAGACCCGCGCCGAGGTGAAGTT
CGAGGGCGACACCCTGGTGAACCGCATCGAGCTGAAGGGCATCGACTTCAAGGAGGACGG
CAACATCCTGGGGCACAAGCTGGAGTACAACTACAACAGCCACAACGTCTATATCATGGC
CGACAAGCAGAAGAACGGCATCAAGGTGAACTTCAAGATCCGCCACAACATCGAGGACG
GCAGCGTGCAGCTCGCCGACCACTACCAGCAGAACACCCCCATCGGCGACGGCCCCGTGC
TGCTGCCCGACAACCACTACCTGAGCTACCAGTCCGCCCTGAGCAAAGACCCCAACGAGA
AGCGCGATCACATGGTCCTGCTGGAGTTCGTGACCGCCGCCGGGATCACTCTCGGCATGGA
CGAGCTGTACAAGTAA

6.2. Protein Sequence

MRGS**HHHHHH**GMADTRIGVTIYKSAGMVSKGEELFTGVVPILVELDGDVNGHKFSVSGEG
EGDATYGKLTLKFICTTGKLPVPWPTLVTTLTWGVQCFSRYPDHMKQHDFFKSAMPEGYVQERT
IFFKDDGNYKTRAEVKFEGDTLVNRIELKGIDFKEDGNILGHKLEYNYISHNVYITADKQKNGIKA
NFKIRHNIEDGSVQLADHYQQNTPIGDGPVLLPDNHYLSTQSALSKDPNEKRDHMVLLEFVTAA
GIGSGTGVGLRKDDAELTAAFNKALGELRQDGTYDKMAKKYFDFNVYGDGGSGGSGGSGGSAL
PETVRIGTDTTYAPFSSKDAKGDFVGFDIDLGNEMCKRMQVKCTWVASDFDALIPSLKAKKIDAII
SSLSITDKRQQEIAFSDKLYAADSRLIAAKGSPIQPTLDSLKGKHVGVLQGSTQEAYANETWRSKG
VDVVAYANQDLVYSDLAAGRLDAALQDEVAASEGFLKQPAGKDFAFAVDELFTGVVPILVELD
GDVNGHKFSVSGEGEGDATYGKLTLKFICTTGKLPVPWPTLVTTFGYGLMCFARYPDHMKQHD
FFKSAMPEGYVQERTIFFKDDGNYKTRAEVKFEGDTLVNRIELKGIDFKEDGNILGHKLEYNYNS
HNVYIMADKQKNGIKVNFKIRHNIEDGSVQLADHYQQNTPIGDGPVLLPDNHYLSYQSALSKDP
NEKRDHMVLLEFVTAAGITLGMDELYK

7. Table S1

Table S1. Overview of the binding parameters of the toolbox sensors.

Sensor	00	0F	0R	F0	FF	FR	R0	RF	RR
R_0	1.70	1.95	2.30	1.52	1.49	1.92	2.20	1.74	2.28
R_{sat}	2.16	2.25	2.37	2.59	2.61	2.52	2.20	3.20	2.87
ΔR	0.46	0.30	0.07	1.07	1.12	0.60	--	1.46	0.59
K_d (μM)	107 ± 5	4.7 ± 0.8	2.5 ± 0.3	67 ± 3	3 ± 0.3	3 ± 0.2	--	81 ± 2	27 ± 4

Includes the FRET-ratios in the non-bound (R_0) state and under saturating conditions (R_{sat}), the sensitivity (ΔR), and the affinity (K_d) of the sensor variants for lysine.

8. Table S2

Table S2. Overview of the binding parameters of the sensor prototype without additional linkers (00) in fresh medium and in culture supernatant (refers to Figure 5 in the main paper).

Measurement System	R_0	R_{sat}	ΔR	K_d	pH
Fresh medium	0.63 ± 0.01	0.84 ± 0.01	0.21	0.37 ± 0.04 mM	7.0
Culture supernatant	0.93 ± 0.02	1.08 ± 0.00	0.15	0.63 ± 0.14 mM	7.5
MOPS buffer	1.70 ± 0.00	2.16 ± 0.00	0.46	107 ± 5 μM	7.3

The mean values of three independent measurements are shown. They were performed directly in the Biolector® cultivation system in the Flowerplates® at 1000 rpm and 30 °C. For comparison, the values measured in MOPS buffer are shown.

9. Setup and Data Analysis of Sensor Application in Microscale Cultivation Experiments of a Lysine Producer

In the following tables, the setup and analysis of the sensor application for L-lysine estimation in microtiter cultivation is explained in detail. See Table S3 for information about the plate layout. The fluorescence signals were measured constantly at λ_{Ex} = 430 ± 5 nm, λ_{Em} = 468 ± 5 nm (ECFP) and λ_{Ex} = 430 ± 5 nm, λ_{Em} = 532 ± 5 nm (Citrine), so the fluorescence intensity in each sample was recorded before each sampling and was subtracted from the measured fluorescence intensity in the presence of the sensor protein (Table S4). With the calculated FRET-ratios of the standards, the mean values and standard deviations of the in-plate calibration respective calibration lines were calculated (Table S5, Figure S5). Based thereon, the respective lysine concentrations in the samples were estimated (Table S6).

10. Table S3

Table S3. Layout of the cultivation plate (Flowerplate®).

<table>
<tr><th rowspan="2">Well</th><th>01</th><th>02</th><th>03</th><th>04</th><th>05</th><th>06</th><th>07</th><th>08</th></tr>
<tr><td colspan="4">Samples</td><td colspan="4">Calibration Standards</td></tr>
<tr><td>A = 0 h</td><td colspan="4" rowspan="6">cultivation of L-lysine producing C. glutamicum DM1933 (4 wells per sample point)</td><td rowspan="6">0 mM L-lysine in-plate calibration</td><td rowspan="6">1 mM L-lysine in-plate calibration</td><td rowspan="6">10 mM L-lysine in-plate calibration</td><td rowspan="6">100 mM L-lysine in-plate calibration</td></tr>
<tr><td>B = 4 h</td></tr>
<tr><td>C = 8 h</td></tr>
<tr><td>D = 12 h</td></tr>
<tr><td>E = 16 h</td></tr>
<tr><td>F = 20 h</td></tr>
</table>

Note: In each row, four wells were filled with culture broth (1–4) and four wells were filled with L-lysine standards for recalibration of the sensors depending on the pH and on the medium composition. Accordingly, the standards in row A were prepared with fresh media, whereas the standards in rows B–F contained increasing amounts of the culture supernatant, which was prepared before with a wild-type strain of *C. glutamicum*. The calibration standards in row B were prepared with a mixture of 67% fresh medium and 33% culture supernatant and accordingly the standards in row C were prepared with a mixture of 33% fresh medium and 67% culture supernatant, whereas rows D, E, and F were prepared with 100% culture supernatant.

11. Table S4

Table S4. Raw data of the fluorescence measurement of the yellow and blue channel at sampling times. In-plate calibration data is shaded in gray, respectively.

**Well Number**	**ECFP-Signal (λ_{Ex} = 430 ± 5 nm, λ_{Em} = 468 ± 5 nm)**			**Citrine-Signal (λ_{Ex} = 430 ± 5 nm, λ_{Em} = 532 ± 5 nm)**			**FRET-Ratio = Citrine/ECFP**
	**Raw Data Before Biosensor Addition**	**Raw Data After Biosensor Addition**	**Fluorescence Signal of the Sensor Minus Background Fluorescence of the Cultivation Broth**	**Raw Data Before Biosensor Addition**	**Raw Data After Biosensor Addition**	**Fluorescence Signal of the Sensor Minus Background Fluorescence of the Cultivation Broth**	
1	0.41	11.38	**10.97**	0.20	7.64	**7.44**	**0.68**
A02	0.35	11.11	**10.75**	0.17	7.61	**7.44**	**0.69**

A03	0.35	11.04	**10.68**	0.18	7.55	**7.37**	**0.69**
A04	0.38	11.11	**10.73**	0.18	7.52	**7.34**	**0.68**
A05	0.37	9.82	**9.45**	0.17	6.57	**6.40**	**0.68**
A06	0.38	9.53	**9.15**	0.17	6.17	**6.00**	**0.66**
A07	0.41	11.38	**10.97**	0.19	9.50	**9.31**	**0.85**
A08	0.65	13.92	**13.27**	0.29	12.08	**11.79**	**0.89**
B01	0.55	12.39	**11.85**	0.40	8.38	**7.98**	**0.67**
B02	0.52	11.98	**11.46**	0.37	8.10	**7.73**	**0.67**
B03	0.55	12.15	**11.60**	0.38	8.34	**7.96**	**0.69**
B04	0.55	12.13	**11.59**	0.40	8.28	**7.88**	**0.68**
B05	0.66	14.13	**13.47**	0.77	9.88	**9.11**	**0.68**
B06	0.69	14.05	**13.37**	0.79	10.07	**9.28**	**0.69**
B07	0.72	14.97	**14.26**	0.81	12.14	**11.33**	**0.79**
B08	0.99	16.84	**15.85**	0.89	14.58	**13.69**	**0.86**
C01	0.95	12.09	**11.14**	0.99	9.08	**8.09**	**0.73**
C02	0.95	11.92	**10.97**	1.00	9.01	**8.01**	**0.73**
C03	0.98	12.32	**11.34**	1.00	9.26	**8.26**	**0.73**
C04	0.99	12.41	**11.42**	1.01	9.43	**8.42**	**0.74**
C05	0.95	16.81	**15.86**	1.20	11.66	**10.46**	**0.66**
C06	0.98	16.66	**15.69**	1.21	12.01	**10.80**	**0.69**
C07	1.06	17.08	**16.02**	1.24	13.81	**12.57**	**0.78**
C08	1.35	19.05	**17.70**	1.33	16.81	**15.48**	**0.87**
D01	1.46	12.50	**11.04**	1.79	10.26	**8.47**	**0.77**
D02	1.46	12.44	**10.98**	1.80	10.31	**8.51**	**0.78**
D03	1.49	12.52	**11.02**	1.83	10.49	**8.66**	**0.79**

D04	1.52	12.72	**11.20**	1.84	10.65	**8.81**	**0.79**
D05	1.38	20.36	**18.98**	1.77	14.45	**12.68**	**0.67**
D06	1.36	20.14	**18.78**	1.76	14.65	**12.89**	**0.69**
D07	1.39	20.20	**18.81**	1.77	16.09	**14.32**	**0.76**
D08	1.62	21.03	**19.41**	1.87	18.27	**16.40**	**0.84**
E01	1.36	13.17	**11.80**	1.96	11.00	**9.04**	**0.77**
E02	1.35	13.07	**11.72**	1.95	11.05	**9.10**	**0.78**
E03	1.40	13.22	**11.82**	1.97	11.20	**9.23**	**0.78**
E04	1.40	13.32	**11.92**	2.00	11.31	**9.31**	**0.78**
E05	1.42	20.78	**19.36**	1.82	14.38	**12.56**	**0.65**
E06	1.40	20.67	**19.26**	1.78	14.81	**13.03**	**0.68**
E07	1.43	20.12	**18.69**	1.80	16.33	**14.53**	**0.78**
E08	1.69	21.41	**19.72**	1.89	18.43	**16.54**	**0.84**
F01	1.43	13.28	**11.85**	2.07	11.01	**8.94**	**0.75**
F02	1.42	13.37	**11.95**	2.06	11.15	**9.09**	**0.76**
F03	1.42	13.38	**11.96**	2.10	11.25	**9.15**	**0.77**
F04	1.48	13.34	**11.86**	2.14	11.26	**9.12**	**0.77**
F05	1.42	20.43	**19.01**	1.79	14.34	**12.55**	**0.66**
F06	1.43	20.67	**19.24**	1.81	14.63	**12.82**	**0.67**
F07	1.39	19.80	**18.41**	1.76	15.52	**13.76**	**0.75**
F08	1.66	20.70	**19.04**	1.87	18.01	**16.14**	**0.85**

12. Table S5

Table S5. FRET-ratios of the in-plate calibration with mean values and standard deviation.

FRET-Ratios of the L-Lysine Standards							Mean Value	Standard Deviation	Deviation in Percent
**Sampling Time**	0 h	4 h	8 h	12 h	16 h	20 h			
0 mM L-lysine	0.68	0.68	0.66	0.67	0.65	0.66	**0.67**	0.01	1.5
1 mM L-lysine	0.66	0.69	0.69	0.69	0.68	0.67	**0.68**	0.01	2.0
10 mM L-lysine	0.85	0.79	0.78	0.76	0.78	0.75	**0.79**	0.03	4.1
100 mM L-lysine	0.89	0.86	0.87	0.84	0.84	0.85	**0.86**	0.02	2.1

Note: the saturation concentration of the sensor with L-lysine under the given conditions is > 10 mM but < 100 mM L-lysine and could not be resolved in the three-point calibration. Therefore, the calibration curves (Figure. S5) apparently increase continuously until 100 mM.

13. Figure S5

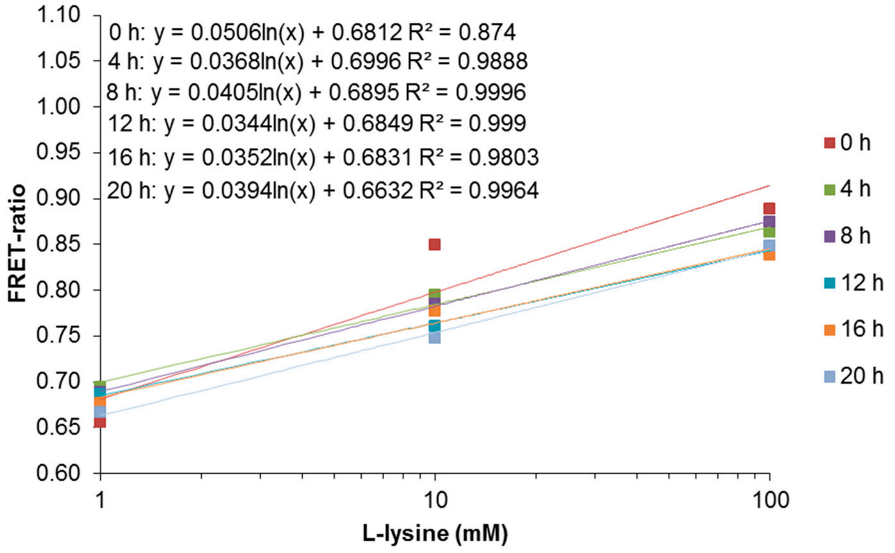

Figure S5. In-plate measured calibration curves.

14. Table S6

Table S6. Analysis of the culture broth using the sensor prototype.

Sampling Time	0 h	4 h	8 h	12 h	16 h	20 h
FRET-ratios of the culture broth						
**Well 1**	0.68	0.67	0.73	0.77	0.77	0.75
**Well 2**	0.69	0.67	0.73	0.78	0.78	0.76
**Well 3**	0.69	0.69	0.73	0.79	0.78	0.77
**Well 4**	0.68	0.68	0.74	0.79	0.78	0.77
**Mean Value**	**0.69**	**0.68**	**0.73**	**0.78**	**0.78**	**0.76**
**Standard Deviation (SD)**	0.01	0.00	0.00	0.01	0.01	0.01
Regression curve: y = a × ln(x) + b						

**a**	0.0506	0.0368	0.0405	0.0344	0.0352	0.0394
**b**	0.6812	0.6996	0.6895	0.6849	0.6831	0.6632
L-lysine in culture broth						
	0–1 ± 0.12 mM	0–1 ± 0.01 mM	3 mM ± 0.01 mM	15 mM ± 0.02 mM	15 mM ± 0.3 mM	12 ± 0.04 mM

References

1. Kremers, G.J.; Goedhart, J.; van Munster, E.B.; Gadella, T.W., Jr. Cyan and yellow super fluorescent proteins with improved brightness, protein folding, and FRET Forster radius. *Biochemistry* **2006**, *45*, 6570–6580.
2. Griesbeck, O.; Baird, G.S.; Campbell, R.E.; Zacharias, D.A.; Tsien, R.Y. Reducing the environmental sensitivity of yellow fluorescent protein. *J. Biol. Chem.* **2001**, *276*, 29188–29194.
